# Sex-related associations between psychoemotional factors and health-related quality of life in patients with chronic low back pain

**DOI:** 10.3389/fpain.2026.1872295

**Published:** 2026-06-30

**Authors:** Mariia Ovdii, Mykola Kondratiuk, Lilia Yaremenko, Maria Prokopiv, Kateryna Potapova, Andrii Borysenko, Olena Lazarieva, Iurii Kuchyn

**Affiliations:** 1 Bogomolets National Medical University, Kyiv, Ukraine; 2 National University of Ukraine on Physical Education and Sport, Kyiv, Ukraine

**Keywords:** anxiety, chronic pain, depression, kinesiophobia, low back pain, quality of life, sex differences, stress

## Abstract

**Introduction:**

Psychoemotional Factors Are Closely Associated With Health-Related Quality Of Life (Hrqol) In Individuals With Chronic Low Back Pain (Clbp). However, It Remains Unclear Whether These Associations Differ Between Women And Men.

**Aim:**

To Evaluate Sex-Stratified Associations Between Psychoemotional Factors And Hrqol Domains In Adults With Chronic Nonspecific Clbp.

**Material and methods:**

This Cross-Sectional Study Included 223 Adults With Chronic Nonspecific Clbp (138 Women And 85 Men) Assessed Before Initiation Of Outpatient Rehabilitation. Pain Intensity, Anxiety, Depression, Perceived Stress, Kinesiophobia, And Hrqol Were Evaluated Using Standardized Questionnaires. Primary Analyses Examined Associations Between Psychoemotional Thresholds And Eight Sf-36 Domains Separately In Women And Men. Secondary Analyses Assessed Severe Pain (Vas ≥7). Exploratory Logistic Regression Models With Sex-By-Variableinteraction Terms Were Additionally Performed. Multiple Testing Was Controlled Using The Benjamini–Hochberg False Discovery Rate Procedure.

**Results:**

The Prevalence Of Clinically Significant Anxiety, Depression, High Perceived Stress, And Kinesiophobia Did Not Differ Significantly Between Women And Men. In Sex-Stratified Analyses, Elevated Psychoemotional Burden In Women Was Primarily Associated With Lower Scores In Vitality, Mental Health, Social Functioning, And General Health. In Men, Associations Involved A Broader Range Of Hrqol Domains, Including Physical Functioning, Role Limitations, Bodily Pain, And General Health. In Secondary Analyses, High Kinesiophobia Was Associated With Severe Pain In Women (Or 9.00, 95% Ci 2.01–40.32; *Q* = 0.033), Whereas No Psychoemotional Indicator Remained Significantly Associated With Severe Pain In Men After Correction For Multiple Testing. However, Exploratory Sex-By-Variableinteraction Analyses Did Not Demonstrate Statistically Robust Interaction Effects After Adjustment For Potential Confounders And Correction For Multiple Testing.

**Conclusions:**

Psychoemotional Factors Were Closely Associated With Hrqol In Adults With Chronic Nonspecific Clbp. Although Sex-Stratified Analyses Revealed Different Patterns Of Associations In Women And Men, Formal Interaction Analyses Did Not Provide Statistically Robust Evidence That These Associations Differed By Sex. The Findings Support The Importance Of Considering Psychoemotional Factors During Assessment And Rehabilitation Planning In Patients With Chronic Nonspecific Clbp And May Help Inform Future Individualized Rehabilitation Approaches.

## Introduction

Low back pain (LBP) is one of the most prevalent global public health problems. Its epidemiological burden continues to increase, posing a serious challenge to modern healthcare systems. According to the World Health Organization (WHO), approximately 80% of the population experience at least one episode of low back pain during their lifetime. In 2020, around 619 million people worldwide were affected by LBP, and projections indicate that this number will rise to approximately 843 million by 2050, largely driven by population growth and global population aging (1, 2). CLBP is a complex, multifactorial condition that arises from the interplay of structural changes, neurophysiological mechanisms, physical limitations, psychological factors, and social determinants. The prevalence of CLBP in the general population ranges from 20% to 40% and is associated with a substantial psychosocial burden, economic costs, functional disability, and a marked reduction in HRQoL (3–7). HRQoL reflects an individual's subjective perception of their physical, psychological, and social functioning in the context of personal goals, expectations, and cultural values. In patients with CLBP, traditional clinical indicators such as pain intensity alone cannot fully capture the multidimensional impact of the condition, underscoring the need for a comprehensive assessment of HRQoL (8). A growing body of evidence supports the key role of psychosocial factors in pain persistence and chronification. Within the biopsychosocial model of CLBP, components such as psychoemotional distress, anxiety, depression, kinesiophobia, pain catastrophizing, and maladaptive cognitive beliefs substantially influence pain perception, behavioral responses, the degree of functional limitation, and rehabilitation outcomes (9–12). Recognizing the importance of these mechanisms, the International Association for the Study of Pain (IASP) designated 2024 as the Global Year for the Study of Sex and Gender Disparities in Pain, emphasizing the need to account for these aspects in both research and the clinical management of patients with pain (13). Despite the growing body of evidence on the role of psychosocial determinants in chronic low back pain, data on sex-specific associations between psychoemotional predictors and individual domains of HRQoL remain limited. A deeper understanding of these relationships is necessary to improve patient stratification and to develop personalized, sex-sensitive rehabilitation strategies. The aim of this study was to evaluate sex-stratified associations between psychoemotional factors and HRQoL domains in adults with nonspecific low back pain. 36-Item Short Form Health Survey (SF-36) in adults with CLBP.

## Material and methods

### Study design

This cross-sectional study was conducted in 2023–2024 at the Rehabilitation Department of the University Clinic of Bogomolets National Medical University. Patients were recruited at the Rehabilitation Department before initiation of the planned outpatient rehabilitation program. The present study was observational and was designed to evaluate baseline biopsychosocial characteristics rather than the effectiveness of rehabilitation interventions or treatment protocols. Adults aged 18–60 years with CLBP. were enrolled. The selected age range was intended to improve clinical homogeneity of the sample and to reduce the influence of advanced age-related degenerative changes and multimorbidity. The inclusion criterion was nonspecific CLBP lasting longer than 3 months. Exclusion criteria included pain localized exclusively to spinal regions other than the lower back, including the cervical or thoracic spine, pain duration of less than 3 months, and evidence of specific spinal pathology.

All participants underwent a standardized biopsychosocial assessment aimed at identifying red flags, yellow flags, and relevant somatic and mental comorbidities (14). All questionnaires and clinical assessments were completed before the initiation of rehabilitation; therefore, the study reflects baseline biopsychosocial characteristics, psychoemotional status, and health-related quality of life prior to rehabilitation exposure.

Information regarding current pain-related medication use was collected during the standardized assessment. Reported medications were categorized into major groups, including nonsteroidal anti-inflammatory drugs (NSAIDs), analgesics, muscle relaxants, antidepressants, and anticonvulsants. Because the study was not designed to evaluate treatment effectiveness, medication use was considered a potential confounding factor and was included as an adjustment variable in exploratory regression analyses. Only participants with complete data for the variables included in the corresponding analyses were retained in each analytical dataset; missing values were not imputed. All participants provided written informed consent before enrolment, and the collected data were de-identified before analysis.

### Assessment methods

- Pain intensity was assessed using the Visual Analog Scale (VAS; 0–10) (15).
- Anxiety and depression were evaluated using the Hospital Anxiety and Depression Scale (HADS), with scores of 11 or higher indicating clinically significant symptoms (16).
- Perceived stress was measured using the 10-item Perceived Stress Scale (PSS-10); scores of 27 or higher indicated high perceived stress (17).
- Kinesiophobia was assessed using the Tampa Scale of Kinesiophobia-11 (TSK-11); scores of 33 or higher indicated a high level of fear of movement (18).
- HRQoL was assessed using the SF-36, which consists of 36 items grouped into eight domains: Physical Functioning (PF), Role Physical (RP), Bodily Pain (BP), General Health (GH), Vitality (VT), Social Functioning (SF), Role Emotional (RE), and Mental Health (MH). Scores were transformed to a 0–100 scale, with higher values indicating better health status. The first four domains form the Physical Component Summary (PCS), and the last four form the Mental Component Summary (MCS) (19).

The Ukrainian-language versions of the HADS (20), PSS-10 (21), TSK-11 (22), and SF-36 (23) used in this study were previously translated, culturally adapted, and validated for application in Ukrainian-speaking populations.

### Statistical analysis

Statistical analysis was performed using Python 3.12 in the Jupyter Notebook 7.0.8 environment, with standard libraries for numerical computation, data processing, statistical analysis, contingency-table analysis, multiple-comparison correction, confidence interval estimation, and regression modeling. Continuous and ordinal variables were summarized as medians and interquartile ranges (IQRs), whereas categorical variables were summarized as counts and percentages. Baseline differences between women and men were assessed using the Mann–Whitney *U*-test for continuous or ordinal variables and the chi-square test or Fisher's exact test, as appropriate, for categorical variables. For categorical prevalence estimates, 95% confidence intervals (95% CI) were reported.

The analytical strategy was defined hierarchically as primary, secondary, and exploratory. The primary analysis focused on associations between psychoemotional indicators and health-related quality-of-life domains measured by the SF-36. Four psychoemotional threshold variables were analyzed: PSS-10 ≥27, HADS-A ≥11, HADS-D ≥11, and TSK-11 ≥33. For each threshold, SF-36 domain scores were compared separately in women and men using the Mann–Whitney *U*-test.

To control for multiple testing, the Benjamini–Hochberg false discovery rate (BH-FDR) procedure was applied. For the primary SF-36 analyses, each FDR family was defined by sex and psychoemotional indicator. Thus, for each psychoemotional indicator (PSS-10, HADS-A, HADS-D, and TSK-11), the eight *p*-values corresponding to the eight SF-36 domains were adjusted together within women and within men. Consequently, eight simultaneous tests constituted one FDR family. This approach was chosen because the SF-36 domains represent correlated dimensions of the same health-related quality-of-life construct and were evaluated simultaneously for a given psychoemotional indicator.

As a secondary clinically interpretable analysis, additional sex-stratified contingency-table comparisons were performed for severe pain, defined as VAS ≥7. These analyses were used to estimate odds ratios (ORs), risk ratios (RRs), 95% confidence intervals, and exact or asymptotic *p*-values, as appropriate. For severe pain analyses, BH-FDR correction was applied separately within each sex across the four psychoemotional thresholds (PSS-10, HADS-A, HADS-D, and TSK-11). These analyses were not treated as the central inferential framework of the manuscript but rather as supportive clinically interpretable extensions of the primary SF-36 domain analysis.

Missing data were assessed for all study variables before analysis. No missing values were observed for demographic characteristics, pain-related variables, or psychoemotional indicators. Missingness was limited to several SF-36 domains and did not exceed 3.6% for any variable. Because the proportion of missing data was low, complete-case analysis was applied for each statistical comparison and regression model without imputation.

In supplementary exploratory analyses, logistic regression models including sex-by-variableinteraction terms were fitted for severe pain to assess whether the associations of psychoemotional indicators differed by sex after accounting for available covariates. Because these models were exploratory and some threshold-positive subgroups were small, they were interpreted cautiously and retained as supplementary rather than core results. Body mass index (BMI), age, pain duration, pain intensity, and reported pain-related medication use were included as covariates in exploratory regression models to reduce potential confounding. A two-sided *p* < 0.05 was considered statistically significant.

The research fully complied with the bioethical and moral requirements of the Helsinki Declaration, the Council of Europe Convention on Human Rights and Biomedicine, WHO regulations, laws of Ukraine and Order of the Ministry of Health of Ukraine No. 281 of November 01, 2000. The study was approved by the Bioethics Committee of the Bogomolets National Medical University No. 192 dated February 24, 2023.

## Results

A total of 223 adults with chronic nonspecific low back pain were included in the study, comprising 138 women (61.9%) and 85 men (38.1%). Baseline demographic, clinical, and psychoemotional and quality-of-life characteristics stratified by sex are presented in Table 1. Data completeness was high across the study dataset. Demographic characteristics, pain-related measures, BMI, and psychoemotional indicators were available for all participants. Missing values were limited to several SF-36 domains and ranged from 0.9% to 3.6%. Therefore, complete-case analysis was used throughout, and no imputation procedures were performed.

**Table 1:** Baseline demographic, clinical, psychoemotional and quality-of-life characteristics of women and men with chronic nonspecific low back pain (Mann–Whitney U).

Variable	Female, n	Female	Male, n	Male	*p*
Age	138	36.5 [22.0; 46.0]	85	37.0 [24.0; 46.0]	0.916
Body mass index (kg/m^2^)	138	23.9 [20.6; 28.4]	85	25.9 [23.6; 28.7]	0.001
Pain intensity (VAS)	138	5.0 [4.0; 6.0]	85	5.0 [4.0; 6.0]	0.112
Pain duration, months	138	6.0 [3.0; 30.0]	85	3.0 [3.0; 36.0]	0.946
PSS-10 score	138	18.0 [14.0; 22.0]	85	20.0 [15.0; 23.0]	0.108
HADS-A score	138	5.0 [2.0; 7.0]	85	5.0 [3.0; 8.0]	0.558
HADS-D score	138	3.0 [1.0; 6.0]	85	2.0 [1.0; 5.0]	0.204
TSK-11 score	138	24.0 [20.0; 27.0]	85	24.0 [20.0; 28.0]	0.746
Physical functioning	136	75.0 [60.0; 90.0]	85	70.0 [45.0; 90.0]	0.031
Role physical	131	100.0 [50.0; 100.0]	84	50.0 [0.0; 100.0]	<0.001
Role emotional	132	100.0 [33.0; 100.0]	84	67.0 [0.0; 100.0]	0.118
Vitality	132	50.0 [35.0; 70.0]	84	50.0 [35.0; 70.0]	0.785
Mental health	132	64.0 [51.0; 80.0]	84	60.0 [44.0; 73.0]	0.385
Social functioning	132	75.0 [62.5; 100.0]	84	75.0 [50.0; 87.5]	0.002
Bodily pain	132	67.5 [55.0; 78.1]	84	55.0 [32.5; 77.5]	0.005
General health	132	55.0 [45.0; 65.0]	84	50.0 [40.0; 60.0]	0.002

Continuous and ordinal variables are presented as median [interquartile range]. VAS, Visual Analog Scale; PSS-10, 10-item Perceived Stress Scale; HADS-A, Hospital Anxiety and Depression Scale, anxiety subscale; HADS-D, Hospital Anxiety and Depression Scale, depression subscale; TSK-11, Tampa Scale of Kinesiophobia-11.

Women and men did not differ significantly with respect to age, pain intensity, pain duration, perceived stress, anxiety, depression, or kinesiophobia, whereas men had a higher BMI. Median age was 35.0 [24.0; 46.0] years in women and 34.0 [28.0; 43.0] years in men (*p* = 0.916). Median pain intensity on the VAS was 5.0 [4.0; 6.0] in women and 5.0 [3.0; 7.0] in men (*p* = 0.112). Pain duration was 12.0 [6.0; 36.0] months in women and 12.0 [6.0; 24.0] months in men (*p* = 0.946). Men had a higher BMI than women: 25.9 [23.6; 28.7] vs. 23.9 [20.6; 28.4] (*p* = 0.001). Median PSS-10 score was 18.0 [14.0; 22.0] in women and 20.0 [15.0; 23.0] in men (*p* = 0.108); median HADS-A score was 5.0 [2.0; 7.0] vs. 5.0 [3.0; 8.0] (*p* = 0.558); median HADS-D score was 3.0 [1.0; 6.0] vs. 2.0 [1.0; 5.0] (*p* = 0.204); and median TSK-11 score was 24.0 [20.0; 27.0] vs. 24.0 [20.0; 28.0] (*p* = 0.746). Women reported higher scores in PF (75.0 [60.0–90.0] vs. 70.0 [45.0–90.0], *p* = 0.031), RP (100.0 [50.0–100.0] vs. 50.0 [0.0–100.0], *p* < 0.001), SF (75.0 [62.5–100.0] vs. 75.0 [50.0–87.5], *p* = 0.002), BP (67.5 [55.0–78.1] vs. 55.0 [32.5–77.5], *p* = 0.005), and GH (55.0 [45.0–65.0] vs. 50.0 [40.0–60.0], *p* = 0.002). No statistically significant sex differences were identified in VT (*p* = 0.785), MH (*p* = 0.385), or RE (*p* = 0.118). These findings indicate that women reported better quality of life in several physical, pain-related, and social domains despite comparable levels of pain intensity and psychoemotional symptoms. The prevalence of clinically significant psychoemotional disturbances is summarized in Table 2.

**Table 2:** Prevalence of clinically significant psychoemotional disturbances in women and men with chronic nonspecific low back pain.

Indicator	Female	Male	Test	*p*
PSS-10 ≥ 27	14/138 (10.1%; 95% CI 6.1–16.3)	9/85 (10.6%; 95% CI 5.7–18.9)	Chi-square	0.916
HADS-A ≥ 11	12/138 (8.7%; 95% CI 5.0–14.6)	12/85 (14.1%; 95% CI 8.3–23.1)	Chi-square	0.204
HADS-D ≥ 11	8/138 (5.8%; 95% CI 3.0–11.0)	3/85 (3.5%; 95% CI 1.2–9.9)	Fisher exact	0.539
TSK-11 ≥ 33	8/138 (5.8%; 95% CI 3.0–11.0)	7/85 (8.2%; 95% CI 4.0–16.0)	Chi-square	0.480

Data are presented as n/N (%; 95% confidence interval). PSS-10, 10-item Perceived Stress Scale; HADS-A, Hospital Anxiety and Depression Scale, anxiety subscale; HADS-D, Hospital Anxiety and Depression Scale, depression subscale; TSK-11, Tampa Scale of Kinesiophobia-11; CI, confidence interval.

**Table 3:** Associations of elevated psychoemotional indicators with severe pain (VAS ≥7), stratified by sex (fisher exact).

Sex	Scale	Outcome	Exposed outcome	Unexposed outcome	OR (95% CI)	RR (95% CI)	*p*	q_BH
Female	PSS-10	VAS ≥7	4/14 (28.6%)	13/124 (10.5%)	3.42 (0.94–12.46)	2.73 (1.03–7.22)	0.073	0.097
	HADS-A		3/12 (25.0%)	14/126 (11.1%)	2.67 (0.64–11.03)	2.25 (0.75–6.74)	0.169	0.169
	HADS-D		3/8 (37.5%)	14/130 (10.8%)	4.97 (1.07–23.07)	3.48 (1.25–9.68)	0.059	0.097
	TSK-11		4/8 (50.0%)	13/130 (10.0%)	9.00 (2.01–40.32)	5.00 (2.11–11.86)	0.008	0.033
Male	PSS-10	VAS ≥7	3/9 (33.3%)	12/76 (15.8%)	2.67 (0.57–12.47)	2.11 (0.73–6.09)	0.192	0.594
	HADS-A		3/12 (25.0%)	12/73 (16.4%)	1.70 (0.39–7.30)	1.52 (0.53–4.34)	0.430	0.574
	HADS-D		1/3 (33.3%)	14/82 (17.1%)	2.43 (0.20–29.09)	1.95 (0.39–9.83)	0.470	0.594
	TSK-11		2/7 (28.6%)	13/78 (16.7%)	2.00 (0.35–11.50)	1.71 (0.49–6.05)	0.599	0.599

Data are presented separately for women and men. Elevated psychoemotional indicators were defined as high perceived stress (10-item Perceived Stress Scale [PSS-10] ≥27), clinically significant anxiety (Hospital Anxiety and Depression Scale, anxiety subscale [HADS-A] ≥11), clinically significant depression (Hospital Anxiety and Depression Scale, depression subscale [HADS-D] ≥11), and high kinesiophobia (Tampa Scale of Kinesiophobia-11 [TSK-11] ≥33). Severe pain was defined as Visual Analog Scale (VAS) ≥7. For each comparison, the proportion of participants with severe pain in the exposed and unexposed groups, odds ratios (ORs), risk ratios (RRs), raw *p*-values, and Benjamini–Hochberg false discovery rate-adjusted q-values are shown. Because several sex-stratified threshold-positive subgroups were small, all comparisons were evaluated using Fisher's exact test and should be interpreted as secondary clinically interpretable analyses.

The proportions of participants exceeding the predefined thresholds for anxiety, depression, perceived stress, and kinesiophobia did not differ significantly between women and men. Clinically significant anxiety (HADS-A ≥11) was present in 12/138 women (8.7%; 95% CI 5.0–14.6) and 12/85 men (14.1%; 95% CI 8.3–23.0) (*p* = 0.204). Clinically significant depression (HADS-D ≥11) was identified in 8/138 women (5.8%; 95% CI 3.0–11.0) and 3/85 men (3.5%; 95% CI 1.2–9.8) (*p* = 0.539). High perceived stress (PSS-10 ≥27) was observed in 14/138 women (10.1%; 95% CI 6.1–16.3) and 9/85 men (10.6%; 95% CI 5.7–18.8) (*p* = 0.916). High kinesiophobia (TSK-11 ≥33) was present in 8/138 women (5.8%; 95% CI 3.0–11.0) and 7/85 men (8.2%; 95% CI 4.1–15.9) (*p* = 0.480). Thus, the main sex-related difference in this cohort was not the prevalence of psychoemotional burden itself, but the pattern of its associations with HRQoL domains. Sex-stratified associations between elevated psychoemotional indicators and severe pain (VAS ≥7), including odds ratios, risk ratios, and confidence intervals, are presented in Table 3.

To account for multiplicity, Benjamini–Hochberg false discovery rate correction was applied separately within each sex × psychoemotional indicator family across the eight SF-36 domains. Therefore, both raw *p*-values and FDR-adjusted q-values are reported. Interpretation of statistically robust findings was based primarily on FDR-adjusted q-values below 0.05.

The results are presented according to the predefined analytical hierarchy. Primary analyses evaluated associations between psychoemotional indicators and SF-36 domains. Secondary analyses examined severe pain (VAS ≥7) as a clinically interpretable outcome. Exploratory analyses assessed sex-by-variableinteraction terms in adjusted logistic regression models.

### Primary analysis: associations between psychoemotional indicators and SF-36 domains

**Figure 1:**
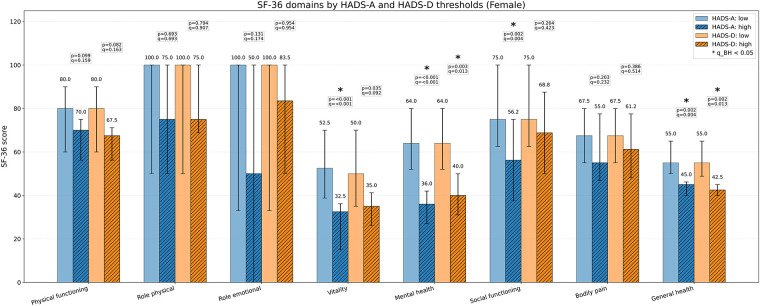
SF-36 domain scores according to anxiety and depression thresholds in women with chronic nonspecific low back pain.

**Figure 2:**
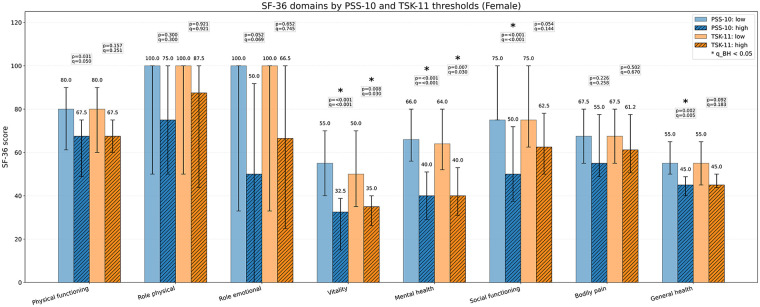
SF-36 domain scores according to perceived stress and kinesiophobia thresholds in women with chronic nonspecific low back pain.

**Figure 3:**
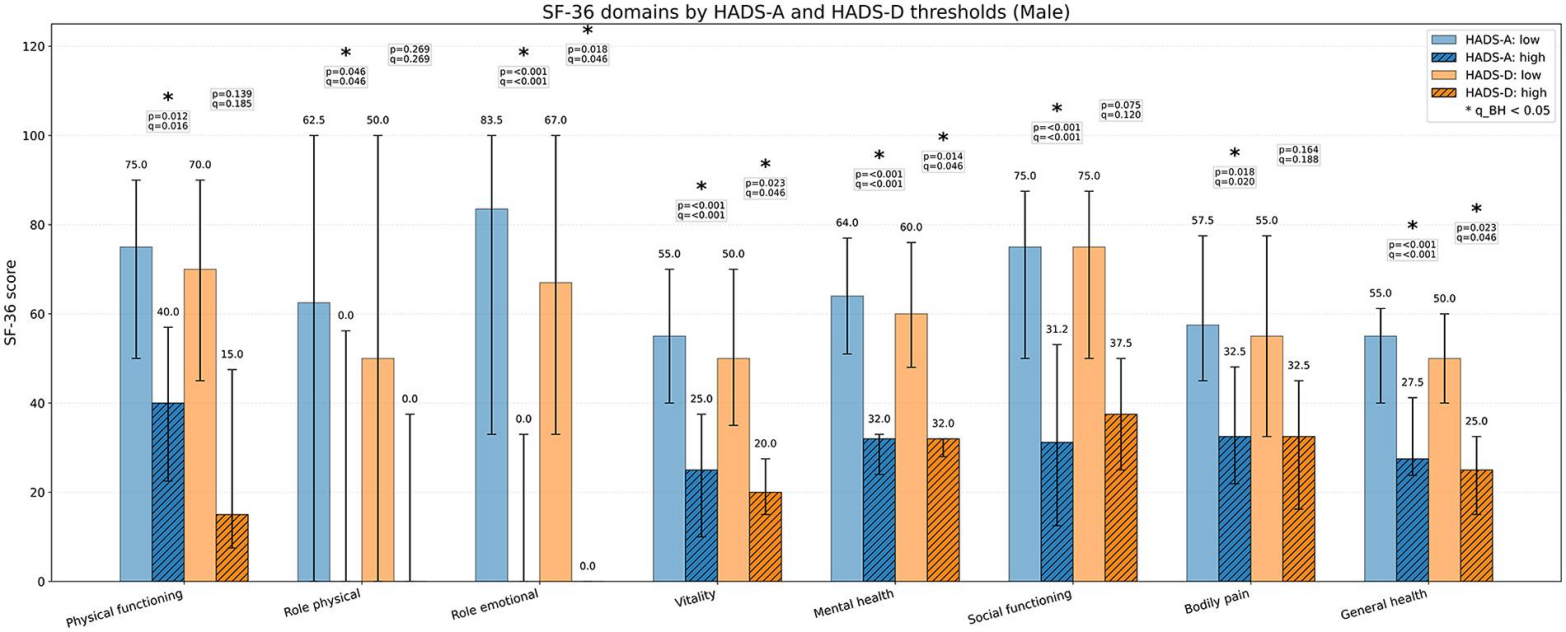
SF-36 domain scores according to anxiety and depression thresholds in men with chronic nonspecific low back pain.

**Figure 4:**
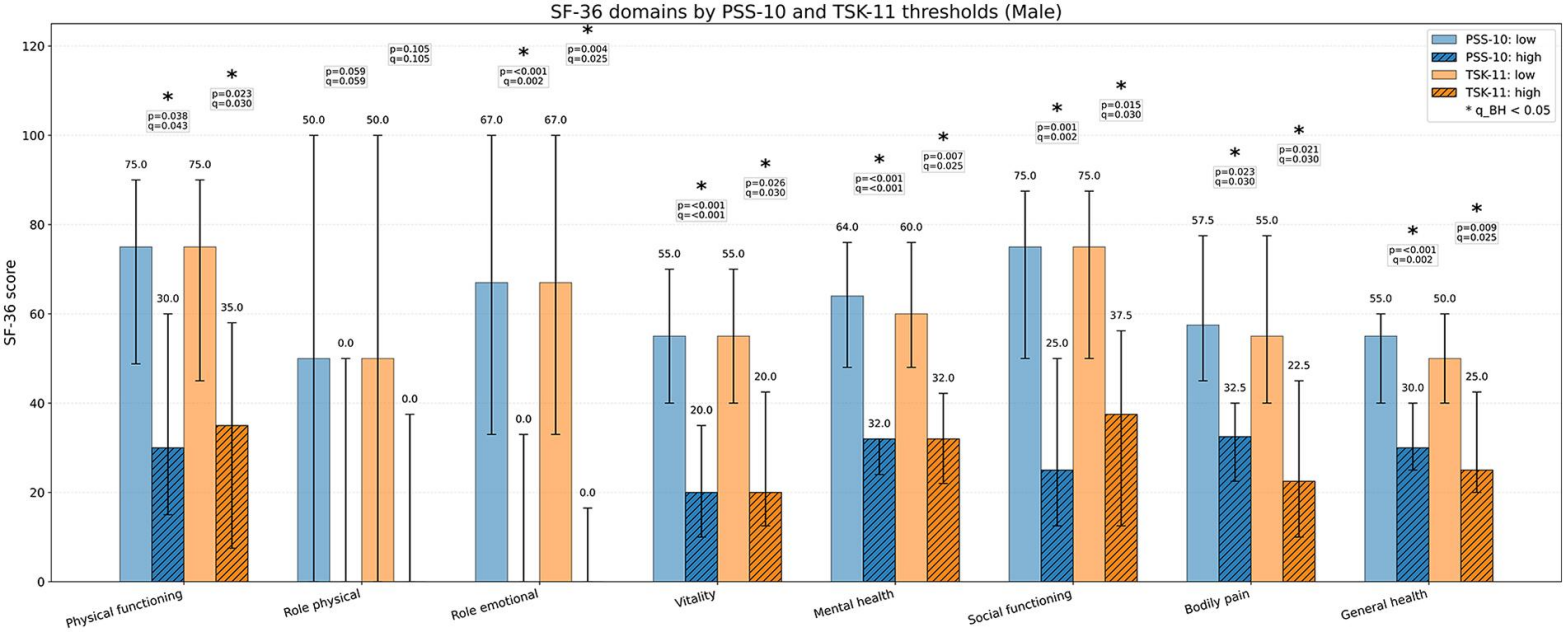
SF-36 domain scores according to perceived stress and kinesiophobia thresholds in men with chronic nonspecific low back pain.

Sex-stratified analyses of the eight SF-36 domains according to psychoemotional thresholds are shown in Figures 1–4, with full numerical results, raw *p*-values.

In women, statistically robust associations were concentrated mainly in VT, MH, SF, and GH. It was found that, in women, statistically significant associations between psychoemotional measures and quality-of-life outcomes were predominantly observed for the SF-36 scales comprising the mental health component. A high level of perceived stress according to the PSS-10 (≥27 points) was significantly associated with lower role limitations due to emotional problems (*p* = 0.031), VT (*p* < 0.001), MH (*p* < 0.001), SF (*p* < 0.001), and GH (*p* = 0.002). Clinically significant anxiety according to the HADS-A (≥11 points) was associated with a significant decrease in VT (*p* < 0.001), SF (*p* = 0.002), GH (*p* = 0.002), and MH (*p* < 0.001). Clinically significant depression according to the HADS-D (≥11 points) was significantly associated with lower VT (*p* < 0.001), MH (*p* = 0.003), and GH (*p* = 0.002). A high level of kinesiophobia according to the TSK-11 (≥33 points) also had a statistically significant negative impact on VT (*p* < 0.001) and the MH component (*p* = 0.007). No statistically significant associations were found in women between psychoemotional measures and the SF-36 domains of PF and BP.

In men, psychoemotional burden was associated with a broader pattern of HRQoL impairment involving both mental and physical domains. Clinically significant anxiety according to the HADS-A (≥11 points) was accompanied by significantly lower scores for PF (*p* = 0.045), role limitations due to physical problems (*p* = 0.012), role limitations due to emotional problems (*p* < 0.001), VT (*p* = 0.022), MH (*p* < 0.001), SF (*p* < 0.001), BP (*p* = 0.018), and GH (*p* < 0.001). In men, a high level of perceived stress according to the PSS-10 (≥27 points) was significantly associated with lower role limitations due to physical health (*p* = 0.037), role limitations due to emotional problems (*p* < 0.001), VT (*p* = 0.034), MH (*p* < 0.001), SF (*p* = 0.001), BP (*p* = 0.022), and GH (*p* < 0.001). Clinically significant depression according to the HADS-D (≥11 points) was significantly associated with lower role limitations due to emotional problems (*p* = 0.018), VT (*p* = 0.007), MH (*p* = 0.013), and GH (*p* = 0.022). A high level of kinesiophobia according to the TSK-11 (≥33 points) also showed a statistically significant negative effect on role limitations due to physical problems (*p* = 0.022) and role limitations due to emotional problems (*p* = 0.004), VT (*p* = 0.026), MH (*p* = 0.007), SF (*p* = 0.015), BP (*p* = 0.021), and GH (*p* = 0.009). Thus, in men, psychoemotional burden showed a broader and more severe domain profile, involving a broader range of SF-36 domains than observed among women.

### Secondary analysis: severe pain

Additional sex-stratified binary-outcome analyses were conducted for severe pain (VAS ≥7) to provide effect-size estimates.

In women, high kinesiophobia (TSK-11 ≥33) was significantly associated with severe pain, with an OR of 9.00 (95% CI 2.01–40.32) and an RR of 5.00 (95% CI 2.11–11.86) (*p* = 0.008, *q* = 0.033). Elevated depressive symptoms showed a borderline association with severe pain (OR 4.97, 95% CI 1.07–23.07; RR 3.48, 95% CI 1.25–9.68; *p* = 0.059, q = 0.097), but did not remain significant after FDR correction. High perceived stress showed OR 3.42 (95% CI 0.94–12.42) and RR 2.73 (95% CI 1.03–7.22) (*p* = 0.073, q = 0.097), and high anxiety showed OR 2.67 (95% CI 0.65–10.91) (*p* = 0.169). These effect-size estimates are presented as secondary clinically interpretable results. In men, none of the psychoemotional indicators remained statistically significant for severe pain after FDR correction. High perceived stress yielded OR 2.67 (95% CI 0.57–12.47) (*p* = 0.192, *q* = 0.594), high anxiety OR 1.70 (95% CI 0.39–7.30) (*p* = 0.430, *q* = 0.574), high depressive symptoms OR 2.43 (95% CI 0.20–29.09) (*p* = 0.470, *q* = 0.594), and high kinesiophobia OR 2.00 (95% CI 0.35–11.50) (*p* = 0.599, *q* = 0.599).

### Exploratory analysis: sex-by-variableinteraction models

These secondary analyses were treated as supportive rather than central. Exploratory logistic regression models including sex-by-variableinteraction terms were fitted for severe pain (VAS ≥7). The models were adjusted for age, BMI, pain duration, and reported pain-related medication use. No statistically significant sex-by-variableinteraction was identified. Specifically, the adjusted sex-by-high-stress interaction yielded OR 0.68 (95% CI 0.08–5.77; *p* = 0.727), the sex-by-clinically significant anxiety interaction OR 0.62 (95% CI 0.07–5.59; *p* = 0.672), the sex-by-clinically significant depression interaction OR 0.52 (95% CI 0.02–11.75; *p* = 0.682), and the sex-by-high-kinesiophobia interaction OR 0.25 (95% CI 0.02–3.02; *p* = 0.278). Adjustment for covariates did not materially alter the overall pattern of findings. These results indicate that, although formal interaction testing was performed, there was no statistically robust evidence that the associations between psychoemotional indicators and severe pain differed by sex. Accordingly, these analyses should be interpreted as exploratory sensitivity analyses rather than as the primary basis for inference.

## Discussion

CLBP is one of the leading contemporary medical and social problems and is widely recognized as a multifactorial condition arising from a complex interplay of biological, psychological, and social factors (24). Patients with this condition consistently demonstrate significantly reduced HRQoL, driven by persistent pain, functional limitations, and associated psychoemotional disturbances (25–29). The aim of this study was to evaluate sex-stratified associations between psychoemotional factors and HRQoL domains in adults with nonspecific CLBP. Importantly, the primary inferential framework of the study was based on SF-36 domain analyses, whereas severe-pain comparisons and interaction models were considered secondary and exploratory, respectively. The study population comprised patients with nonspecific CLBP undergoing outpatient rehabilitation. Most participants were of working age, and women constituted the majority of the sample, which is consistent with global epidemiological evidence indicating a higher burden of this condition among females (30, 31). This pattern may be related to biological, psychological, and social factors that differ between women and men, including hormonal influences, pain modulation mechanisms, and psychoemotional characteristics (32–35). An interesting finding of the present study was that women and men did not differ significantly in pain intensity, pain duration, perceived stress, anxiety, depression, or kinesiophobia. Nevertheless, men reported significantly poorer quality of life in several SF-36 domains, particularly those related to physical health, including PF, RP, BP and GH. These findings suggest that the burden of CLBP may be reflected differently in everyday functioning between sexes, even when pain severity and psychoemotional distress appear comparable. These findings contrast with the majority of the literature, which typically reports poorer psychoemotional status among women with CLBP (36, 37). One possible explanation for these findings is the presence of sex-specific responses to chronic pain. Despite similar levels of pain intensity, psychological distress, and kinesiophobia, men reported poorer physical health-related quality of life, suggesting that chronic pain may have a greater impact on everyday functioning in men than in women. This discrepancy may reflect differences in behavioral adaptation and coping strategies. Men may be more likely to respond to persistent pain by restricting physical activity, reducing participation in daily tasks, and adopting less adaptive coping behaviors, which may contribute to poorer physical functioning and greater role limitations despite comparable symptom severity.

In addition, the sociocultural context of the study population should be considered. The ongoing war in Ukraine may represent an additional source of stress for men, particularly those of working and military age, due to concerns related to mobilization, occupational responsibilities, and prolonged uncertainty. Although these factors were not directly assessed, they may have amplified the negative impact of chronic pain on perceived health and physical functioning. Collectively, these findings suggest that sex differences in HRQoL among individuals with CLBP may be influenced not only by pain-related and psychoemotional factors but also by differences in behavioral adaptation and contextual stressors. However, sex-stratified analyses revealed different patterns of associations between psychoemotional factors and SF-36 domains in women and men. In men, clinically significant anxiety and high perceived stress were associated with lower scores across both mental and physical HRQoL domains, including PF, RE, RF, and BP. These findings may reflect a closer coexistence of psychoemotional distress and poorer functioning among men in this cohort, although the mechanisms underlying this pattern cannot be determined from the present cross-sectional data. In women, high levels of stress, anxiety, depression were predominantly associated with lower MH, VT, and SF, whereas the physical SF-36 domains remained relatively stable. Such a profile may be consistent with a greater concentration of associations within psychosocial domains of HRQoL among women. Associations involving kinesiophobia also showed different domain patterns in women and men: in women, they were concentrated mainly in psychoemotional domains, whereas in men they extended to PF, pain-related measures, and GH. These findings are consistent with previous studies reporting associations between psychoemotional factors and lower HRQoL among patients with chronic pain (38, 39). The observed sex-stratified patterns may be useful for informing future individualized assessment and rehabilitation planning; however, they should not be interpreted as evidence of confirmed sex-specific effects. Our previous studies (40) have shown that, within the Ukrainian population, men with CLBP generally exhibit more pronounced lifestyle-related risk factors for chronic pain compared to women. This finding points to the possible influence of these factors on patients' psychological and emotional state and highlights the importance of applying sex-specific approaches in the modern treatment of CLBP. The results of this study confirm that chronic pain is a multifactorial phenomenon and, at the same time, a highly individual experience shaped by multiple contextual factors (41, 42). Psychosocial factors play a key role in shaping the pain experience, emphasizing the need for their timely assessment and the implementation of sex-sensitive rehabilitation strategies. Within the rehabilitation program, we implemented an educational module focused on training patients in pain coping strategies. The primary aim of the module is to increase awareness of the mechanisms of chronic pain and to develop self-management competencies. The observed differences in association patterns may be useful for informing future development of individualized educational components within rehabilitation programs. However, the present findings should not be interpreted as evidence of confirmed sex-specific effects because formal interaction analyses did not demonstrate statistically robust sex-by-variableinteractions.

The influence of treatment-related factors should be interpreted in the context of the study design. All assessments were performed before initiation of the rehabilitation program; therefore, the observed associations reflect baseline patient characteristics rather than rehabilitation-related effects. Information regarding current pain-related medication use was available and was included as an adjustment variable in exploratory models. However, detailed information regarding medication dosage, treatment duration, adherence, previous treatment history, and specific rehabilitation modalities was not systematically collected.

Several limitations should be acknowledged. First, the cross-sectional design precludes causal inference. Second, although current pain-related medication use was available and considered in adjusted exploratory analyses, detailed information regarding medication dosage, treatment duration, and adherence was not collected. Third, all assessments were performed before rehabilitation initiation; therefore, the study was not designed to evaluate the effects of rehabilitation interventions. Fourth, several threshold-positive subgroups were relatively small, resulting in wide confidence intervals and limited precision of some effect estimates, particularly in secondary and exploratory analyses.

## Conclusion

No statistically significant sex differences were observed in the prevalence of clinically significant anxiety, depression, high perceived stress, or kinesiophobia among adults with nonspecific CLBP. However, sex-stratified analyses revealed different patterns of associations between psychoemotional factors and HRQoL domains in women and men.

Among women, elevated psychoemotional burden was predominantly associated with lower scores in mental and social domains of the SF-36. Among men, these associations extended to a broader range of domains, including physical functioning, role limitations, pain-related domains, and mental health-related domains. In secondary analyses, high kinesiophobia was associated with severe pain in women, whereas no psychoemotional indicator remained significantly associated with severe pain in men after correction for multiple testing.

Nevertheless, exploratory sex-by-variable interaction analyses did not provide statistically robust evidence that the observed associations differed by sex after adjustment for age, BMI, pain duration, pain intensity, medication use, and correction for multiple testing. Therefore, the observed sex-stratified patterns should be interpreted as descriptive associations rather than as confirmed sex-specific effects.

Overall, the findings highlight the importance of considering psychoemotional factors when evaluating patients with nonspecific CLBP and suggest that these factors are closely associated with HRQoL. The observed association patterns may be useful for informing individualized assessment and rehabilitation approaches. Because of the cross-sectional design, the findings should be interpreted as associative rather than causal. Future longitudinal studies with larger and more balanced samples are required to confirm the descriptive sex-stratified association patterns observed in the present study and to determine whether these associations have prognostic or clinical significance over time.

## Data Availability

The raw data supporting the conclusions of this article will be made available by the authors, without undue reservation.
